# Changes in the Distribution of Membrane Lipids during Growth of Thermotoga maritima at Different Temperatures: Indications for the Potential Mechanism of Biosynthesis of Ether-Bound Diabolic Acid (Membrane-Spanning) Lipids

**DOI:** 10.1128/AEM.01763-21

**Published:** 2022-01-25

**Authors:** Diana X. Sahonero-Canavesi, Laura Villanueva, Nicole J. Bale, Jade Bosviel, Michel Koenen, Ellen C. Hopmans, Jaap S. Sinninghe Damsté

**Affiliations:** a Department of Marine Microbiology and Biogeochemistry (MMB), NIOZ Royal Netherlands Institute for Sea Research, Den Burg, The Netherlands; b Utrecht University, Faculty of Geosciences, Department of Earth Sciences, Utrecht, The Netherlands; Kyoto University

**Keywords:** membrane-spanning lipids, diabolic acid, biosynthesis, *Thermotoga maritima*, growth phase, ether lipids, *Thermotoga*, lipid biosynthesis

## Abstract

Membrane-spanning lipids are present in a wide variety of archaea, but they are rarely in bacteria. Nevertheless, the (hyper)thermophilic members of the order *Thermotogales* harbor tetraester, tetraether, and mixed ether/ester membrane-spanning lipids mostly composed of core lipids derived from diabolic acids, C_30_, C_32_, and C_34_ dicarboxylic acids with two adjacent mid-chain methyl substituents. Lipid analysis of Thermotoga maritima across growth phases revealed a decrease of the relative abundance of fatty acids together with an increase of diabolic acids with independence of growth temperature. We also identified isomers of C_30_ and C_32_ diabolic acids, i.e., dicarboxylic acids with only one methyl group at C-15. Their distribution suggests they are products of the condensation reaction but are preferably produced when the length of the acyl chains is not optimal. Compared with growth at the optimal temperature of 80°C, an increase of glycerol ether-derived lipids was observed at 55°C. Our analysis only detected diabolic acid-containing intact polar lipids with phosphoglycerol (PG) head groups. Considering these findings, we hypothesize a biosynthetic pathway for the synthesis of membrane-spanning lipids based on PG polar lipid formation, suggesting that the protein catalyzing this process is a membrane protein. We also identified, by genomic and protein domain analyses, a gene coding for a putative plasmalogen synthase homologue in T. maritima that is also present in other bacteria producing *sn*-1-alkyl ether lipids but not plasmalogens, suggesting it is involved in the conversion of the ester-to-ether bond in the diabolic acids bound in membrane-spanning lipids.

**IMPORTANCE** Membrane-spanning lipids are unique compounds found in most archaeal membranes, but they are also present in specific bacterial groups like the *Thermotogales*. The synthesis and physiological role of membrane-spanning lipids in bacteria represent an evolutionary and biochemical open question that points to the differentiation of the membrane lipid composition. Understanding the formation of membrane-spanning lipids is crucial to solving this question and identifying the enzymatic and biochemical mechanism performing this procedure. In the present work, we found changes at the core lipid level, and we propose that the growth phase drives the biosynthesis of these lipids rather than temperature. Our results identified physiological conditions influencing the membrane-spanning lipid biosynthetic process, which can further clarify the pathway leading to the biosynthesis of these compounds.

## INTRODUCTION

Membrane lipids play a fundamental role in forming the physical barrier surrounding the cells, the cytoplasmic membrane. In *Bacteria*, membrane lipids are commonly composed of glycerol, derived from glycerol-3-phosphate (G3P), attached via ester bonds to fatty acid molecules, forming a bilayer structure. On the other hand, the opposite stereoisomer of glycerol, derived from glycerol-1-phosphate and linked to isoprenoid alkyl chains via ether bonds, is characteristic for the membrane lipids of *Archaea* (1, 2). Most of the archaeal lipid membranes are organized as monolayers of lipids spanning the membrane (i.e., membrane-spanning lipids), formed of isoprenoidal tetraether lipids called glycerol dialkyl glycerol tetraethers (GDGTs), thought to be more rigid and impermeable than the bilayer membranes from bacteria and eukaryotes (3). However, some bacterial groups also possess membranes organized as monolayers that contain lipids resembling the archaeal GDGT lipids but with the bacterial G3P stereochemistry and comprised of branched alkyl chains rather than isoprenoidal chains.

The membrane-spanning C_30_ (13,14-dimethyloctacosanedioic acid) and C_32_ (15,16-dimethyltriacontanedioic acid) diabolic acids (DAs; from Greek *diabollo*) (4) have been observed in members of the order *Thermotogales* as part of their core lipids (CL; without the polar headgroup) (5–7). Other C_29_ to C_32_ diacids, i.e., with methyl groups on the C-13 and C-16 carbons, have been isolated and characterized from the thermophilic anaerobic eubacterium *Thermoanaerobacter* spp. (8, 9). Nonisoprenoidal GDGTs have also been detected in peats (10), but their source is not fully constrained, although recent studies have confirmed that specific *Acidobacteria* subdivisions produce membrane-spanning lipids with the 13,16-dimethyl octacosanedioic acid (or *iso*-diabolic acid) as a building block (11).

**FIG 1:**
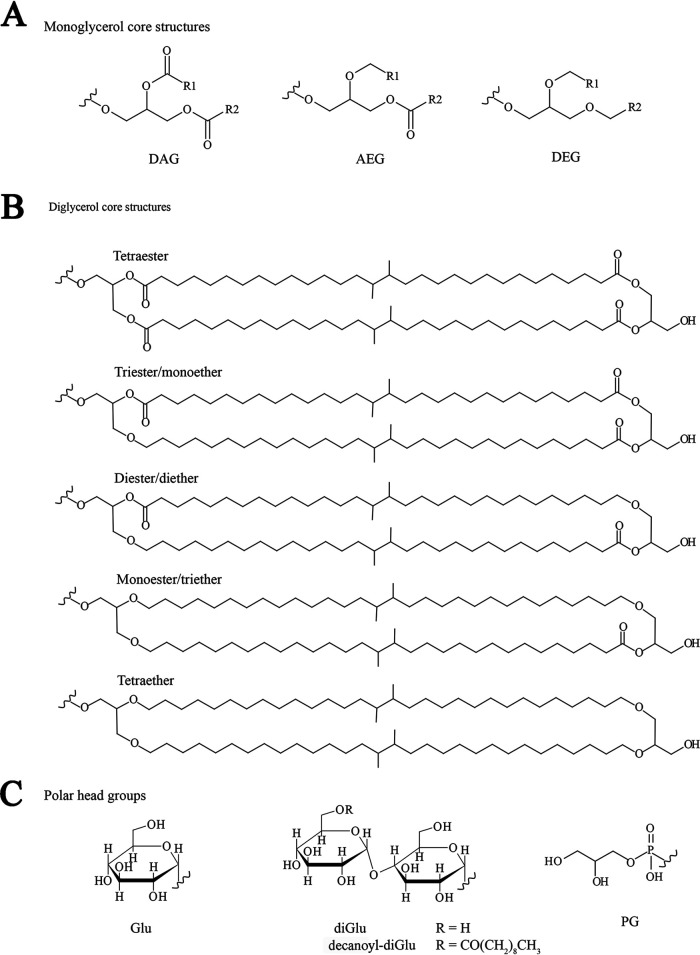
Structures of membrane-spanning and non-membrane-spanning IPLs found in Thermotoga maritima. (A) General structures of non-membrane spanning IPLs. R1 and R2 are alkyl chains. (B) Examples of possible structures of membrane-spanning IPLs. (C) Some of the detected polar head groups. Glu, glucosyl; di-Glu, diglucosyl; PG, phosphoglycerol that can be attached to the structures shown in panels A and B.

Previous reports on bacteria from the order of *Thermotogales* have described the core lipid composition composed of straight-chain fatty acids (FAs) (predominantly C_14_ and C_16_) as well as the C_30_ and C_32_ DAs (5, 6), which are arranged as tetraesters, mixed ether-ester compounds, or tetraethers (7), and detected diacylglycerol lipids bearing unusual polar head groups (glycolipid 1 and glycolipid 2; Fig. 1 shows the structure) (12). Although these studies have provided an excellent overview of the membrane lipid composition itself, changes in the relative distribution of the membrane lipids due to membrane lipid adaptation to physiological conditions remain to be explored. Furthermore, it remains unclear which intact polar lipids (IPLs; i.e., including the polar head group) contain DAs in members of the *Thermotogales*.

From an evolutionary perspective, the formation of membrane-spanning lipids in bacteria is an open question and concerns the evolutionary divergence of the lipid composition, or lipid divide, of all cellular forms of life, as these membrane lipids have mixed archaeal and bacterial traits that could enlighten how this division occurred (2, 13, 14). To tackle this question, the enzymatic steps leading to the formation of the bacterial membrane-spanning lipids need to be elucidated. The molecular mechanism that leads to the biosynthesis of membrane-spanning lipids in bacteria involves the condensation of two FA molecules (15, 16). Although the enzymatic activity catalyzing this coupling mechanism has been detected using extracts of intact or disrupted cells of anaerobic bacteria (16, 17) or other (*iso*) diabolic acid producers (16), the enzyme(s) involved remains unknown. Likewise, the bacterial enzymes responsible for the transformation of the ester bond between the glycerol and DA into an ether bond have not been elucidated. To constrain the missing pieces of the puzzle leading to the formation of bacterial membrane-spanning lipids, physiological studies involving factors that could affect the membrane lipid composition are required.

Here, we identify specific physiological conditions resulting in differences in lipid membrane composition of a membrane-spanning lipid producer of the bacterial order of *Thermotogales*, T. maritima. The hyperthermophilic bacterium T. maritima can grow from 55°C up to 90°C with an optimum at 80°C (18). We determined the composition of core and intact polar membrane lipid of T. maritima at optimal (80°C) and suboptimal (55°C) temperature across growth phases, which resulted in differences in cell morphology at the different temperatures independent of similarities in lipid composition. Based on these findings and on genomic analyses, we identified a potential homologue of the gene involved in the formation of ether bonds in the diabolic acids of T. maritima, and we propose a hypothetical pathway for the biosynthesis of these membrane-spanning lipids.

## RESULTS

Cultivation experiments with T. maritima at optimal (80°C) and suboptimal (55°C) growth temperatures were performed in batch cultures, and lipid and microscopic analysis was performed at different (early exponential, exponential, and stationary) growth phases.

### Core membrane lipid composition.

Gas chromatography-mass spectrometry (GC-MS) analysis of the lipids released after base hydrolysis of intact cells revealed C_14_, C_16_, and C_18_ FAs and C_30_ and C_32_ DAs, as well as glycerol monoethers C_16_ (1-O-hexadecyl glycerol), C_30_ (13,14dimethyl-28-glyceryloxyoctadecanoic acid), and C_32_ (15,16-dimethyl-30-glyceryloxytriacontanoic acid), likely derived from ether/ester mixed species (Table 1), based on their mass spectral characteristics (7).

**TABLE 1:** Relative distribution (percentage of total) of membrane-spanning and non-membrane-spanning core lipids in T. maritima^*a*^

Lipid	Result by growth temp/growth stage					
	80°C			55°C		
	E. Exp	Exponential	Stationary	E. Exp	Exponential	Stationary
Relative distribution (% of total)						
Non-membrane-spanning						
C_14_ FA	2.2 ± 0.2	3.0 ± 0.5	4.4 ± 0.2	1.8 ± 0.1	2.9 ± 1.1	4.1 ± 1.6
C_16_ FA	42.6 ± 4.2	33.4 ± 5.2	22.4 ± 2.1	46.4 ± 6.5	45.9 ± 0.2	21.1 ± 2.8
C_18_ FA	12.8 ± 2.3	2.8 ± 0.4	1.3 ± 0.1	15.0 ± 5.6	5.6 ± 5.1	1.7 ± 0.1
1-O-hexadecyl glycerol (C_16_ FA-GE)	1.1 ± 0.0	0.6 ± 0.3	0.3 ± 0.2	3.3 ± 0.5	3.2 ± 0.8	0.4 ± 0.0
Membrane-spanning						
Diabolic acids						
13,14-Dimethyloctacosanedioic acid (C_30_ DA)	1.3 ± 0.3	1.6 ± 0.2	3.2 ± 0.6	0.5 ± 0.5	1 ± 0.2	2.5 ± 0.1
15,16-Dimethyltriacontanedioic acid (C_32_ DA)	28.3 ± 0.4	42.1 ± 5.5	46.0 ± 6.3	13.4 ± 11.7	23 ± 2.1	38.8 ± 4.3
13,14-Dimethyl-28-glyceryloxyoctadecanoic acid (C_30_ DA-GE)	0.5 ± 0.2	0.8 ± 0.1	1.7 ± 0.9	1 ± 0.6	1.5 ± 0.9	2.9 ± 0.1
15,16-Dimethyl-30-glyceryloxytriacontanoic acid (C_32_ DA-GE)	6.2 ± 2.0	8.1 ± 0.9	10.8 ± 5.1	14.9 ± 1.5	12.5 ± 2.9	18.4 ± 0.3
Monomethyl diacids						
15-Methylnonacosanedioic acid (C_30_ MM)	0.8 ± 0.1	1.1 ± 0.1	2.2 ± 0.5	0.3 ± 0.3	0.5 ± 0	1.6 ± 0.1
15-Methylhentriacontanedioic acid (C_32_ MM)	2.3 ± 0	4.4 ± 0.6	4.8 ± 0.8	0.8 ± 0.8	1.5 ± 0	3.7 ± 0.5
15-Methyl-29-glyceryloxynonadecanoic acid (C_30_ MM-GE)	0.3 ± 0.1	0.6 ± 0.1	1.2 ± 0.7	0.6 ± 0.3	0.9 ± 0.6	2.0 ± 0.1
15-Methyl-31-glyceryloxyhentriacontanoic acid (C_32_ MM-GE)	1.0 ± 0.2	0.9 ± 0.2	1.2 ± 0.6	1.2 ± 0.1	1.0 ± 0.2	2.1 ± 0.0
						
Relative distribution (%, on molar basis)						
Ethers (sum)	4.3	5.0	6.6	10.4	9.2	11.5
Esters (sum)	95.7	95.0	93.4	89.6	90.8	88.5
Membrane spanning (sum)	41.0	59.4	70.5	32.0	41.2	71.2
Non-membrane-spanning (sum)	59.0	40.6	29.5	68.0	58.8	28.8

a E. Exp, early exponential phase. Molar mass changes (%) of ether compounds and membrane-spanning lipids are calculated on the flame ionization detector response to the number of carbon atoms of the derivatized compounds (methyl ester and trimethylsilyl groups).

**FIG 2:**
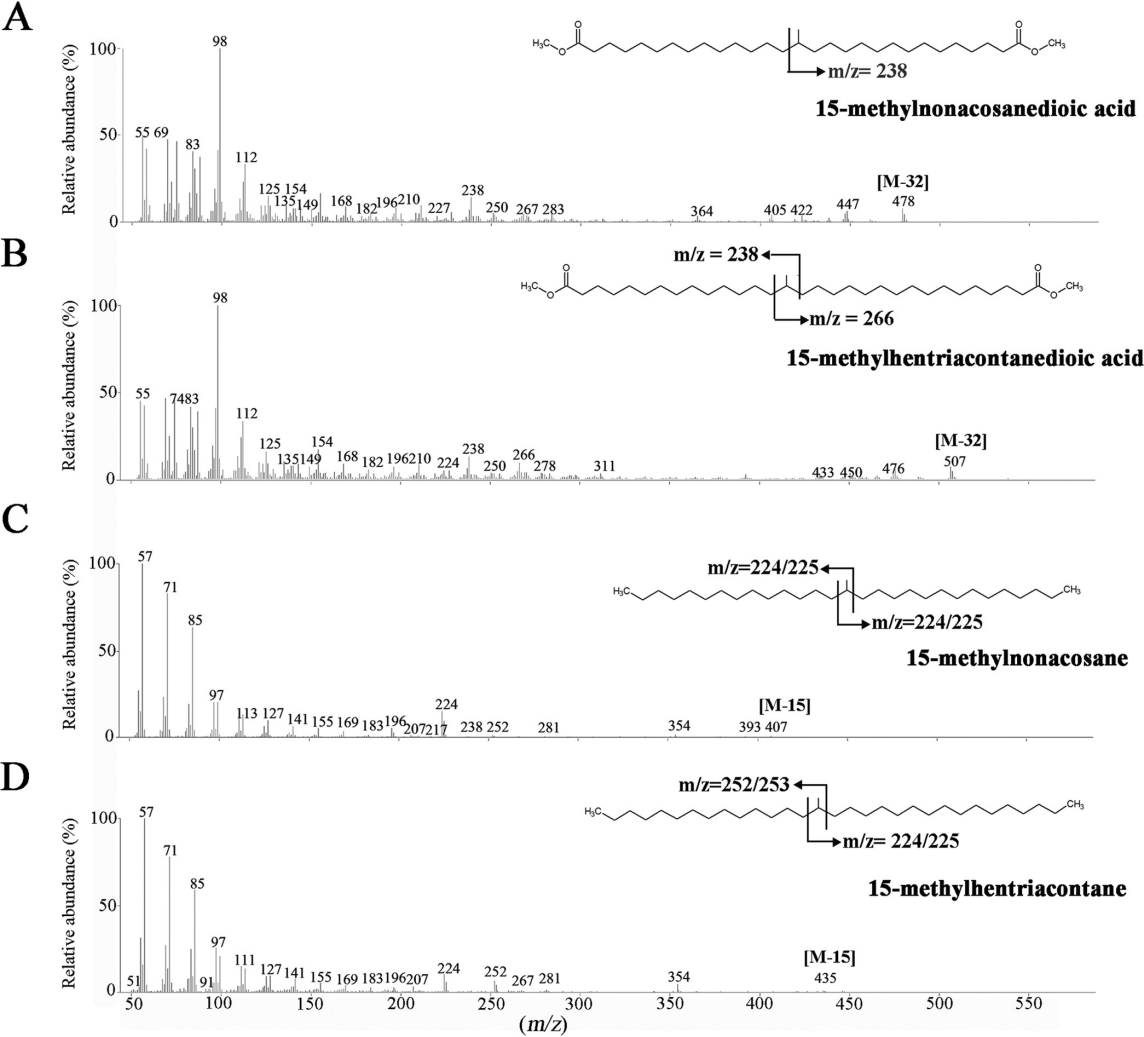
Mass spectra of membrane-spanning monomethyl diacids methyl ester derivatives and the hydrocarbons derived from these diacids after subsequent treatment with LiAlH_4_ and HI/H_2_. (A) 15-Methylnonacosanedioic acid dimethyl ester. (B) 15-Methylhentriacontanedioic acid dimethyl ester. (C) 15-Methylnonacosane. (D) 15-Methylhentriacontane.

**FIG 3:**
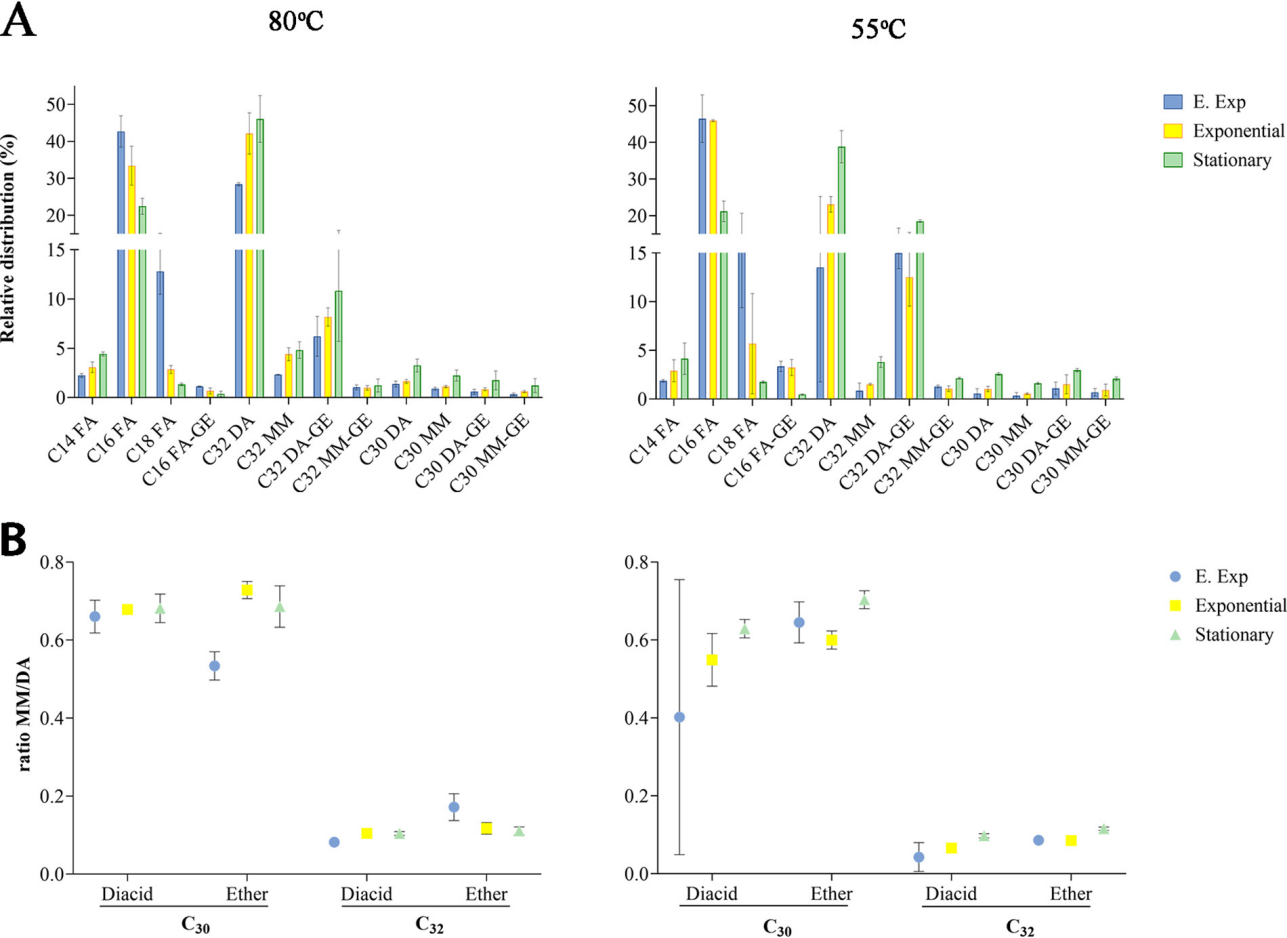
Core lipid composition of T. maritima at optimal (80°C) or suboptimal (55°C) growth temperature during various growth phases. (A) Core lipid distribution of fatty acids (C_14_, C_16_, and C_18_ FA), diabolic acids C_30_ DA (13,14-dimethyloctacosanedioic acid) and C_32_ DA (15,16-dimethyltriacontanedioic acid), fatty acids (ether) C_16_ FA-GE, 1-O-hexadecyl glycerol, diabolic acids glycerol ether C_30_ DA-GE (13,14-dimethyl-28-glyceryloxyoctadecanoic acid), and C_32_ DA-GE (15,16-dimethyl-30-glyceryloxytriacontanoic acid), monomethyl diacids C_30_ MM (15-methylnonacosanedioic acid) and C_32_ MM (15-methylhentriacontanedioic acid), and monomethyl diacids glycerol ether C_30_ MM-GE (15-methyl-29-glyceryloxynonadecanoic acid) and C_32_ MM-GE (15-methyl-31-glyceryloxyhentriacontanoic acid). Bars represent the mean fractional abundance. The error indicated shows the standard deviation from three biological replicates. ANOVA significant statistical differences (*P* < 0.0001) between the early exponential and the stationary phases on the C_16_ and C_32_ lipids across growth phases at each temperature condition are shown. (B) The monomethyl/dimethyl diacid ratio and their glycerol ether derivatives as a function of number of carbon atoms of the diacid and growth temperature and phase. E. Exp, early exponential.

Isomers of the C_30_ and C_32_ DAs and the C_30_ and C_32_ DA glycerol monoethers possessing a similar mass spectrum eluted approximately 0.4 to 0.6 min later from the corresponding DA or DA glycerol monoethers. These isomers have been recognized before in cultures of the *Thermotogales* (7). Careful inspection of the mass spectrum of the isomer eluting after the C_30_ DA suggested that it represents a C_30_ dicarboxylic acid dimethyl ester with only one methyl group at C-15 (Fig. 2A). To confirm this, the diacids were converted to diols by treatment with LiAlH_4_ and then to hydrocarbons by treatment with HI and subsequent hydrogenation. Indeed, the mass spectrum of the hydrocarbon eluting after 13,14-dimethyloctacosane was consistent with 15-methylnonacosane, characterized by a relatively strong *m/z* 224 fragment ion in its mass spectrum (Fig. 2C). Its measured Kovats retention index (RI; 2,933) is in good agreement with the calculated RI value (2,930) (19, 20). Hence, the C_30_ diacid isomer was assigned as a 15-methylnonacosanedioic acid. Similarly, the C_32_ diacid isomer was assigned as a 15-methylhentriacontanedioic acid. The mass spectrum of its dimethyl ester derivative (Fig. 2B) revealed characteristic fragment ions at *m/z* 238 and 266. The mass spectrum of the corresponding hydrocarbon showed relatively strong fragment ions at *m/z* 224 and 252 (Fig. 2D), and the measured RI (3,130) is in good agreement with the calculated RI value (3,130) for 15-methylhentriacontane. For the C_32_ diacids, monomethyl diacid occurred only in relatively low abundance at optimal growth temperature; the ratio of the monomethyl and dimethyl diacids was ca. 0.1 (Fig. 3B, Table 1). Remarkably, however, this ratio for the C_30_ dimethyl and monomethyl was much higher, at ca. 0.7, during all analyzed growth phases. A similar observation was made for the corresponding glycerol derivatives with a ratio of 0.1 for the C_32_ and 0.6 for the C_30_ diacids (Fig. 3B). Similar ratios were observed when cells were grown at suboptimal growth temperatures (Fig. 3B, Table 1).

### Distribution of lipid components across growth phases.

**FIG 4:**
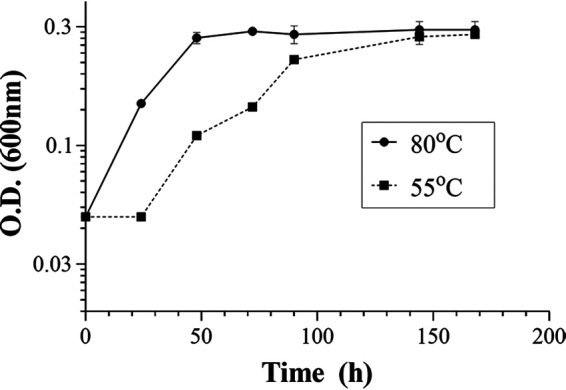
Growth of T. maritima MSB8 in basal medium at either suboptimal (55°C) or optimal (80°C) growth temperature. Growth phases were identified based on OD_600_ measurements. Early exponential (0.1), exponential (0.2), and stationary (0.3 to 0.4) phases are shown. T. maritima was grown at 80°C (●) or 55°C (■).

The growth of T. maritima was monitored at the optimal growth temperature (80°C) with a doubling time of approximately 19 h (Fig. 4). The distribution of the FA and glycerol monoethers changed across growth phases (Fig. 3A). The dominant FA was C_16_, accounting for 43% of the total lipids in the early exponential phase, decreasing to 33% during the exponential phase and 22% by the stationary phase (Table 1). The C_14_ FA increased from 2.2 to 4.4%, and the C_18_ content decreased from 13 to 1.3% between the early exponential and the stationary phase. Conversely, the major C_32_ diacids increased from 28% in the early exponential phase to 42% in the exponential phase and 46% in the stationary phase, and the C_30_ diacids increased from 1.3 to 3.2% between the early exponential and stationary phases. The corresponding C_32_ and C_30_ monomethyl diacids also increased from 2.3 to 4.8% and from 0.8 to 2.2%, respectively, between the early exponential and stationary phases.

The glycerol ether compounds consisted of the C_16_ glycerol monoether, which decreased from 1.1 to 0.3% across growth phases, and the C_30_ and C_32_ DA monoethers. The relative abundance of these components increased from 0.5 to 1.7% and 6% to 11%, respectively, while the corresponding monomethyl diacid C_30_ and C_32_ monoethers also increased from 0.3 to 1.2% and from 1.0 to 1.2% at the same growth interval (Table 1).

The intact polar lipid (IPL) composition of T. maritima grown under optimal conditions (80°C) was analyzed from freeze-dried cells harvested at three different growth phases, resulting in the detection and identification of a range of IPLs (Table 2; Fig. 1 shows structures). A number of the IPLs were non-membrane-spanning, including phosphatidylglycerols (PGs) with a diacylglycerol (DAG) core (C_16_/C_16_) and two PGs with a dietherglycerol (DEG) core (C_16_/C_14_ and C_16_/C_16_). Their fractional abundance decreased from 2.4 to 0.1% for the DAG core and increased from 1.9% to 8% for the DEG core across the three growth phases (Table 2). Additionally, PGs with a mixed acyl-etherglycerol (AEG) core (C_16_/C_14_ and C_16_/C_16_) were detected; their sum decreased from 6.9 to 0.9%. A range of lipids with dihexose head groups was detected (Table 2). These are presumably diglucosyls (diGlu) based on previous detailed analysis of the glycolipids of T. maritima using nuclear magnetic resonance (NMR) spectroscopy (12). Four diGlus with DAG cores (C_16_/C_14_, C_16_/C_16_, C_16_/C_18_, and C_18_/C_18_) decreased from 14.8 to 10.2% across the three growth phases, while the abundance of a single diGlu with an AEG core (C_16_/C_16_) showed little change (i.e., from 1.0 to 2.8%). A minor IPL with a monohexose head group was assigned as a monoglucosyl (Glu) with a DAG core (C_16_/C_16_) and increased slightly in abundance from 0.3 to 0.5%. Additionally, four IPLs with an unusual polar head group, a decanoyl-diGlu described previously in T. maritima by Manca et al. (12) (Fig. 1), were detected (see Table S1 in the supplemental material for accurate mass of fragments). Two decanoyl-diGlus had C_16_/C_14_ and C_16_/C_16_ DAG cores and their sum increased from 7.3 to 8.7% over the growth phases, and two had C_16_/C_14_ and C_16_/C_16_ AEG cores and their sum increased modestly from 5.9 to 8.3% (Table 2). Membrane-spanning IPLs were also detected but only with PG head groups. Fifteen different diglycerols, with a C_30_/C_30_, C_30_/C_32_, and C_32_/C_32_ core, ranging in their ether/ester composition between tetraether and tetraester, were identified (Table 2). All the PGs with a tetraether C_30_/C_30_, C_30_/C_32_, and C_32_/C_32_ core, triether/monoester, monoether/triester C_30_/C_30_, C_30_/C_32_, and diether/diester C_30_/C_30_, C_30_/C_32_ cores increased in their fractional abundance from 15.6 to 25.3%. Those with monoether/triester, tetraester, and diether/diester containing the C_32_/C_32_ core decreased in their fractional abundance from 43.2 to 32.6% (Table 2) over the three growth phases. C_30_ and C_32_ DAs attached to a glycerol moiety also were detected, and their fractional abundance increased from 0.5 to 2.5% with time (Table 2). Potentially these compounds are formed from the membrane-spanning IPLs by loss of the head group.

**TABLE 2:** Relative abundance and mass spectral characteristics of intact polar lipids in T. maritima

IPL composition		Mass spectral characteristics^*a*^				Relative abundance^*b*^ (%) by growth temp/growth stage^*c*^					
						80°C			55°C		
Polar head group^*d*^	Core lipid^*e*^	Dominant ion	Mass (*m/z*)	AEC^*f*^	Δmmu^*g*^	E. Exp	Exp	Sta	E. Exp	Exp	Sta
Non-membrane-spanning (sum)						40.6	42.7	39.6	62.6	49.8	39.9
PG	DAG, C_32:0_	[M + NH_4_]^+^	740.543	C_38_H_79_NO_10_P	0.7	2.4	2.9	0.1	7.5	1.7	0.0
PG	AEG, C_30:0_	[M + H]^+^	681.506	C_36_H_74_O_9_P	0.6	1.0	0.2	0.3	0.6	1.9	0.1
PG	AEG, C_32:0_	[M + H]^+^	709.537	C_38_H_78_O_9_P	0.5	5.9	2.2	0.6	8.4	7.2	0.2
PG	DEG, C_30:0_	[M + H]^+^	667.527	C_36_H_76_O_8_P	0.3	0.4	1.1	2.3	0.0	0.5	2.9
PG	DEG, C_32:0_	[M + H]^+^	695.559	C_38_H_80_O_8_P	0.2	1.5	4.0	5.7	0.3	1.8	6.2
diGlu	AEG, C_32:0_	[M+NH_4_]^+^	896.667	C_47_H_94_NO_14_	0.3	1.0	2.8	2.8	1.8	1.8	3.2
diGlu	DAG, C_30:0_	[M + NH_4_]^+^	882.615	C_45_H_88_NO_15_	0.3	0.6	1.2	2.0	2.5	1.2	1.8
diGlu	DAG, C_32:0_	[M + NH_4_]^+^	910.646	C_47_H_92_NO_15_	0.2	7.2	9.1	6.9	22.0	11.2	7.3
diGlu	DAG, C_34:0_	[M + NH_4_]^+^	938.678	C_49_H_96_NO_15_	0.5	4.1	2.3	1.1	6.2	2.0	1.9
diGlu	DAG, C_36:0_	[M + NH_4_]^+^	966.709	C_51_H_100_NO_15_	0.5	2.9	0.3	0.2	1.1	0.3	0.3
Glu	DAG, C_32:0_	[M + NH_4_]^+^	748.594	C_41_H_82_NO_10_	0.7	0.3	0.7	0.5	0.1	0.1	0.9
decanoyl-diGlu	DAG, C_32:0_	[M + NH_4_]^+^	1,064.783	C_57_H_110_NO_16_	0.6	6.8	6.8	6.4	7.1	10.0	5.9
decanoyl-diGlu	DAG, C_30:0_	[M + NH_4_]^+^	1,036.751	C_55_H_106_NO_16_	0.6	0.5	1.7	2.3	0.1	0.8	2.3
decanoyl-diGlu	AEG, C_32:0_	[M + NH_4_]^+^	1,050.803	C_57_H_112_NO_15_	0.5	5.6	6.0	6.4	5.0	8.7	5.1
decanoyl-diGlu	AEG, C_30:0_	[M + NH_4_]^+^	1,022.772	C_55_H_108_NO_15_	1.0	0.3	1.3	1.9	0.1	0.6	1.9
Membrane-spanning (sum)						59.4	57.3	60.4	37.4	50.2	60.1
PG	Tetraether, C_64:0_	[M + H]^+^	1,232.076	C_73_H_148_O_11_P	0.4	0.1	0.9	0.6	0.0	0.0	1.3
PG	Triether/monoester, C_64:0_	[M + NH_4_]^+^	1,263.082	C_73_H_149_NO_12_P	0.8	1.4	3.6	2.9	0.1	0.8	5.0
PG	Diether/diester, C_64:0_	[M + NH_4_]^+^	1,277.060	C_73_H_147_NO_13_P	0.1	12.0	9.5	11.9	1.9	9.1	10.8
PG	Monoether/triester, C_64:0_	[M + NH_4_]^+^	1,291.040	C_73_H_145_NO_14_P	0.2	20.3	11.3	12.6	12.6	18.8	12.8
PG	Tetraester, C_64:0_	[M + NH_4_]^+^	1,305.020	C_73_H_143_NO_15_P	0.6	10.9	16.3	8.1	18.9	10.7	7.7
PG	Tetraether, C_62:0_	[M + H]^+^	1,204.044	C_71_H_144_O_11_P	0.4	0.1	0.7	0.5	0.0	0.0	1.1
PG	Triether/monoester, C_62:0_	[M + NH_4_]^+^	1,235.051	C_71_H_145_NO_12_P	0.8	0.8	2.3	1.7	0.1	0.3	2.9
PG	Diether/di ester, C_62:0_	[M + NH_4_]^+^	1,249.028	C_71_H_143_NO_13_P	1.1	3.5	4.5	6.0	0.2	2.3	5.9
PG	Monoether/triester, C_62:0_	[M + NH_4_]^+^	1,263.008	C_71_H_141_NO_14_P	0.4	6.1	2.7	6.5	1.4	5.3	4.0
PG	Tetraester, C_62:0_	[M + NH_4_]^+^	1,276.988	C_71_H_139_NO_15_P	0.7	2.7	2.9	4.4	2.1	2.1	3.3
PG	Tetraether, C_60:0_	[M + H]^+^	1,204.044	C_69_H_140_O_11_P	0.7	0.0	0.1	0.1	0.0	0.0	0.2
PG	Triether/monoester, C_60:0_	[M + NH_4_]^+^	1,235.051	C_69_H_141_NO_12_P	0.8	0.1	0.3	0.2	0.0	0.0	0.4
PG	Diether/di ester, C_60:0_	[M + NH_4_]^+^	1,249.028	C_69_H_139_NO_13_P	1.0	0.1	0.2	0.3	0.0	0.1	0.2
PG	Monoether/triester, C_60:0_	[M + NH_4_]^+^	1,263.008	C_69_H_137_NO_14_P	0.2	0.4	0.2	1.1	0.0	0.3	0.6
PG	Tetraester, C_60:0_	[M + NH_4_]^+^	1,276.988	C_69_H_135_NO_15_P	0.3	0.3	0.3	1.0	0.0	0.2	0.6
Glycerol	Diabolic acid C_32:0_	[M + NH_4_]^+^	570.546	C_35_H_72_NO_4_	0.3	0.3	1.0	1.6	0.0	0.3	1.9
Glycerol	Diabolic acid C_30:0_	[M + NH_4_]^+^	542.514	C_33_H_68_NO_4_	0.1	0.2	0.5	0.9	0.0	0.0	1.2

a Determined by HRMS analysis of extract from 80°C culture harvested at stationary phase.

b Determined by ITMS analysis.

c E. Exp, early exponential phase; Exp, exponential phase; Sta, stationary phase.

d PG, phosphoglycerol; diGlu, diglucosyl; Glu, glucosyl.

e DEG, diether glycerol; AEG, acyl ether glycerol; DAG, diacyl glycerol.

f AEC, Assigned elemental composition.

g mmu, milli-mass unit; Δmmu = (measured mass −calculated mass) × 1,000.

Cultivation of T. maritima at suboptimal temperature (55°C) resulted in a doubling time of approximately 32 h (Fig. 4). As observed at optimal growth temperature, the relative distribution of FA and DA core lipids changed significantly across growth phases (Fig. 3A). The fractional abundance of the C_16_ FA accounted for 46% at the early exponential phase and decreased to 21% in the stationary phase (Table 1). The C_14_ FA increased gradually from 2% to 4% while the C_18_ FA decreased gradually from 15% to 2% across growth phases. The relative abundance of the C_30_ diacids increased from 0.5 to 2.5%, while the C_32_ diacids increased from 13% to 23% and increased up to 39% during the batch cultivation. The fractional abundance of the corresponding C_32_ and C_30_ monomethyl dicarboxylic acids also increased across growth phases from 0.8 to 3.7% and from 0.3 to 1.6%, respectively. The fractional abundance of the C_16_ glycerol monoether decreased from 3.4 to 0.4%, whereas those of the C_30_ and C_32_ diacid glycerol monoethers increased from 1% to 3% and from 15% to 19%, respectively, from the early exponential to stationary phase of growth. The fractional abundance of the corresponding monomethyl C_30_ and C_32_ monoethers also increased from 0.6 to 2.0% and from 1.2 to 2.1% at the same growth interval (Table 1, Fig. 3A).

The same IPL species as those observed under the optimal growth conditions (80°C) were detected in T. maritima at suboptimal growth conditions (55°C). The fractional abundance of non-membrane-spanning PG-DAGs decreased, from 8% to 0%, as did the PG-AEGs, from 9 to 0.3%, while the fractional abundance of the PG-DEGs increased across the three growth phases to the same extent as the observed at 80°C, from 0.3% to 9% (Table 2). The fractional abundance of the four diGlu-DAGs was much higher at the early exponential phase but decreased substantially, i.e., from 32% to 11%. The single diGlu-AEG increased, from 1.8 to 3.2%, as did the Glu-DAG, from 0.1 to 0.9%. The fractional abundance of the membrane-spanning PGs with tetraether, triether/monoester, triester/monoether, and diether/diester C_30_/C_30_, C_30_/C_32_, and C_32_/C_32_ cores increased substantially, from 18% to 49%, while that of the membrane-spanning PGs with a tetraester C_32_/C_32_ core decreased from 19% to 8% (Table 2) over the three growth phases. Finally, the C_30_ and C_32_ diacids attached to a glycerol moiety were not detected at the first growth stage, but fractional abundance was ca. 3% at the end of the growth curve, a scale increase similar to that seen at 80°C.

### Cell morphology and membrane integrity.

**FIG 5:**
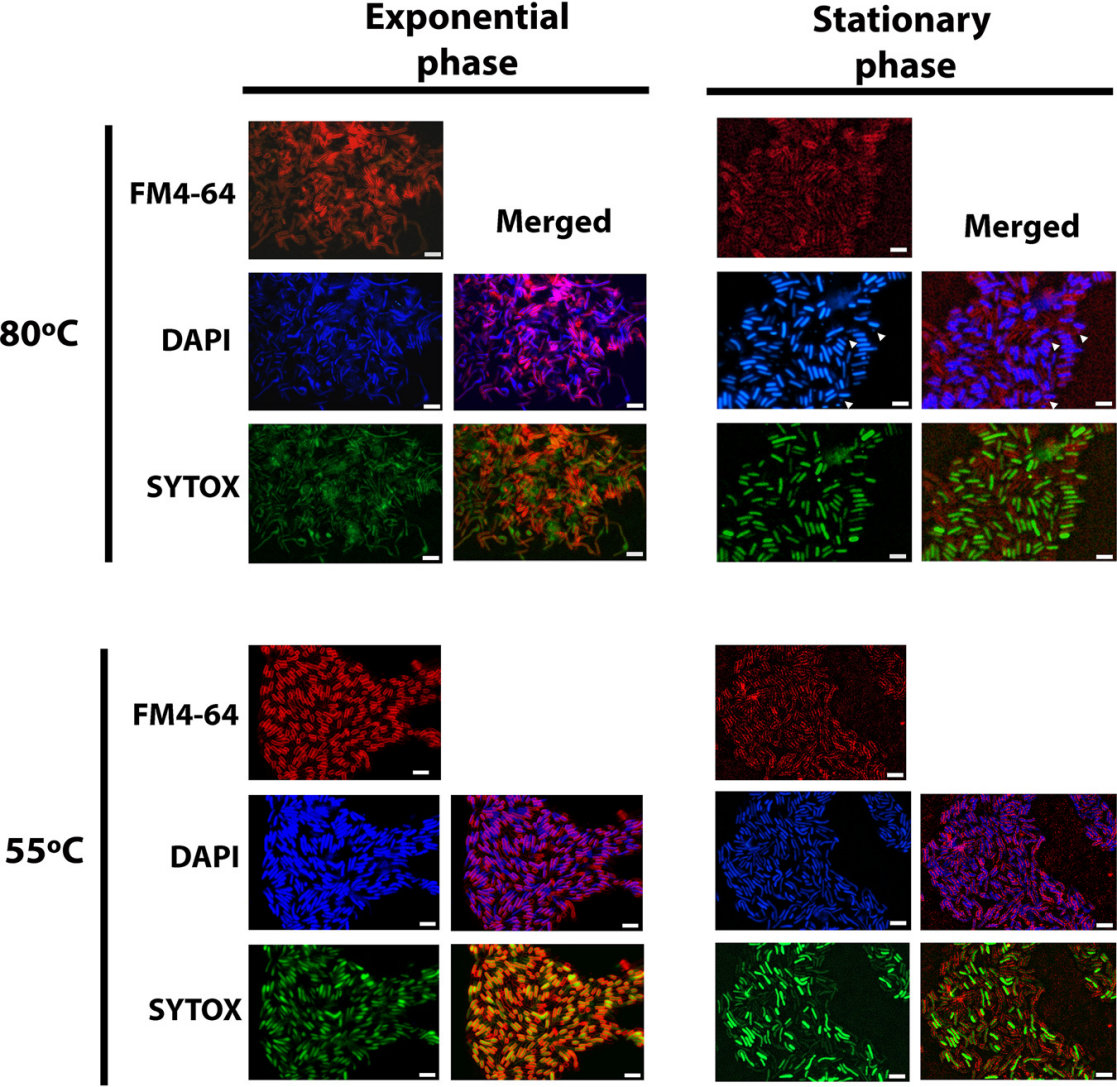
Fluorescence microscopy of Thermotoga maritima at optimal (80°C) and suboptimal (55°C) temperature growth conditions. T. maritima cells from exponential and stationary phases at optimal temperature and exponential- and stationary-phase cells grown at suboptimal temperature are shown. Cellular profiles were imaged after the selection of the appropriate filter for each specific stain. Membrane stain was FM4-64 (red). DNA stains were membrane permeable, DAPI (blue) membrane-impermeable, and SYTOX (green). Scale bar, 5 μm. The arrows indicate coccoid cells.

To determine changes in the morphology and the membrane integrity of T. maritima cells associated with changes in the membrane lipid composition, we used fluorescence microscopy and image analysis on cells collected at the exponential and stationary phase. A specific membrane dye (FM4-64) allowed the visualization of cell lipid boundaries and morphologies (Fig. 5), and the stained DNA (4′,6-diamidino-2-phenylindole dihydrochloride [DAPI]) was utilized to quantitatively measure various morphological cell parameters (area, length, and circularity). To analyze the cell membrane permeability properties, fluorescence intensity values were calculated from imaged cells simultaneously stained with membrane-permeable (DAPI) and membrane-impermeable (SYTOX green) DNA dyes.

Cells visualized with the membrane dye FM4-64 showed the inner and outer membranes combined in one strong signal surrounding the edges of the cell. At the exponential phase, T. maritima culture was composed mostly of cells with a rod shape (Table 3). Two morphologies were detected at the stationary phase, being rod-shaped (Fig. 5) and coccoid cells. The latter were mostly detected during prolonged stationary phase (Fig. S1). Cells from stationary phase showed a significant decrease in the area from 1.3 to 0.9 μm^2^, from 2.3 to 1.8 μm in length, and increased their circularity values from 0.5 to 0.6 compared to the exponential-phase cells (Table 3, Fig. S2). The detected fluorescent signal for the DNA dyes showed no significant changes between the growth phases (Table 3, Fig. S2).

**TABLE 3:** Cell measurements of cellular morphological properties of T. maritima^*a*^

Temp (^o^C) and growth phase	Cell measurement from:				
	DAPI				SYTOX green intensity (AU)
	Area (μm^2^)	Length (μm)	Circularity	Intensity (AU)	
80					
Exponential	1.29 ± 0.23	2.36 ± 0.35	0.53 ± 0.01	67.4 ± 7.8	42.0 ± 4.6
Stationary	0.90 ± 0.13	1.81 ± 0.16	0.60 ± 0.02	66.5 ± 15.6	39.0 ± 1.2
55					
Exponential	3.01 ± 0.23	4.14 ± 0.21	0.45 ± 0.03	65.2 ± 4.8	35.3 ± 6.2
Stationary	2.23 ± 0.32	3.52 ± 0.29	0.43 ± 0.03	80.8 ± 8.0	61.0 ± 16.3

a Data represent the mean ± SD, *n* = 3 independent images with 100 cells measured on each image. AU, arbitrary units.

Cells grown at 55°C and visualized with the membrane dye FM4-64 showed a fluorescent signal belonging to the inner and outer membrane, mainly distributed over the cell ends, forming the toga's membranous structure surrounding the cytoplasmic membrane (Fig. 5). Bacterial cell profiles at the exponential phase were dominated by elongated rods, and at the stationary phase, the main morphology detected was the rod-shaped bacteria (Fig. 5, Table 3). Cells from stationary phase showed a significant decrease in area from 3 to 2.2 μm^2^, from 4.1 to 3.5 μm in length, and with no significant changes in their circularity values compared to the exponential-phase cells (Table 3, Fig. S2). The detected fluorescent signal for both DNA dyes showed significant increased values, from 65 to 81 arbitrary units (AU) of intensity for DAPI and from 35 to 61 AU for SYTOX green within the growth phases (Table 3, Fig. S2).

## DISCUSSION

### Dynamic biosynthesis of membrane-spanning lipids across growth phases.

In this study, we confirmed the presence of previously reported core lipids (Table 1), identified new lipids (Tables 1 and 2), and extended our analyses to the core lipid composition at optimal and suboptimal growth temperatures in T. maritima across growth phases. A previous study on the stable carbon isotopic fractionation associated with the biosynthesis of FAs in T. maritima across growth phases showed a decrease in the relative abundance of the C_16_ FA concomitant with an increase of higher-molecular-weight lipids that were speculated to be DAs (21). Our core lipid analysis provides compelling evidence that the C_16_ FAs are utilized as building blocks for the synthesis of C_32_ DAs at both optimal and suboptimal temperature growth conditions. Statistical analysis (ANOVA) showed the relative abundance of C_16_ FA and C_32_ DA to be significantly different (*P* < 0.0001) between the early exponential and stationary phases with a marked decrease of the relative abundance of the C_16_ FA and a concomitant increase of the relative abundance of the membrane-spanning C_32_ (Fig. 3A). This strongly supports the direct relationship between substrate and product across growth phase, independent of growth temperature.

The core lipid analysis also revealed that the lipid composition at the stationary phase is dominated by DAs (Fig. 3A) with an overall degree of membrane-spanning lipids, increasing from 41% to 71% at optimal growth temperature and from 32% to 71% at suboptimal temperature (Table 1). In their natural environment, bacterial cells are predominantly found in the stationary phase or limited growth rather than exponential phase (22, 23), undergoing changes in morphology and cell physiology (24, 25). Based on the high proportion of C_32_ DAs compared to the C_16_ FAs in the stationary phase, we therefore hypothesize that the membrane-spanning DAs probably play an essential role in determining the physical properties of the membrane in natural T. maritima communities and their fitness and survival.

A former labeling experiment of the DA producer Butyrivibrio fibrisolvens (15) with [16-^2^H_3_]-palmitic acid (C_16_) and [14-^2^H_2_]-palmitic acid demonstrated that the C_32_ DA is biosynthesized by a condensation reaction between two C_16_ FAs at the ω-1 position. This must be induced by abstraction of a hydrogen at C-15, indicating that this most likely is an enzyme-mediated reaction, as the resulting DA consists of only one stereoisomer (15). A C_30_ DA would likewise be biosynthesized by a similar condensation of a C_14_ and a C_16_ FA. In our analysis of T. maritima lipids, we identified the monomethyl C_30_ and C_32_ diacids. Their molecular structure indicates that instead of being formed by condensation of the two ω-1 carbon atoms of the FA precursors, these compounds are likely produced by condensation of ω-1 and the ω position of the two FAs. The ratio of the C_30_ mono- and dimethyl diacids was ca. 0.67 at both growth temperatures (Fig. 3B, Table 1), suggesting only a slight preference for the ω-1/ω-1 condensation. Conversely, for the C_32_ diacids this ratio is ca. 0.1 (i.e., 7- to 8-fold lower; Fig. 3B, Table 1). A similar trend was observed for the monoether-bound diacids (Fig. 3B, Table 1). Based on this observation and the relatively low abundance of the C_30_ compounds, we hypothesize that the enzymatic condensation leading to a membrane-spanning lipid is specifically designed for the ω-1/ω-1 condensation of two C_16_ alkyl chains. With one shorter (C_14_) chain instead of the two C_16_ chains, the condensation is less specific and results in much more by-product. Note that despite the presence of some C_18_ FAs (Table 1), we never identified DAs based on C_18_ alkyl chains in T. maritima lipids.

The enzymatic activity mediating this reaction has been confirmed in membrane enrichments and cell extracts of *Butyrivibrio* and *Sarcina* (17). Although this specific carbon-carbon bond formation between the membrane FAs depends on enzymatic activity, the enzyme or the biosynthetic complex has not been yet identified.

**FIG 6:**
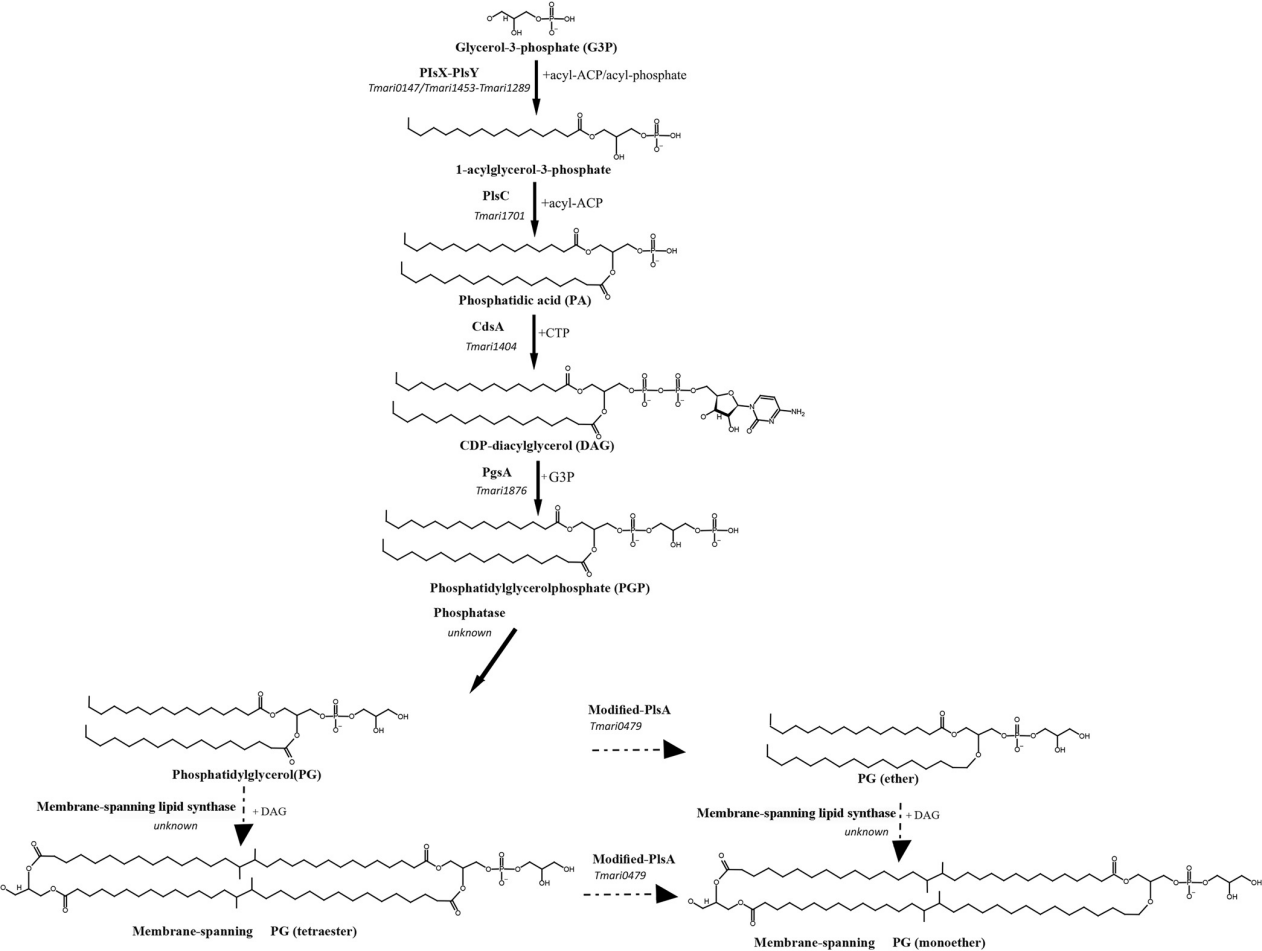
Hypothetical biosynthetic pathway for the synthesis of diabolic acid in T. maritima. This pathway first involves the formation of a PG glycerophospholipid precursor. The genes encoding the enzymes involved in the glycerolipid backbone's biosynthesis were identified in the T. maritima genome, as indicated in the text. The final step leading to PG might be catalyzed by a potential (yet unknown) phosphatase. A membrane-spanning lipid synthase (unknown) must be responsible for joining the ω-1 carbon atoms of the FAs (C_16_) precursors and forming the membrane-spanning compounds. Ether lipids (*sn*-1 position) could be formed from PG or from membrane-spanning PG precursors by a modified *pls*A gene-coding enzyme.

The IPL analysis revealed a range of the membrane-spanning core lipids derived from C_30_/C_30_, C_30_/C_32_, and C_32_/C_32_ diglycerol compounds. Interestingly, all the C_30_/C_30_, C_30_/C_32_, and C_32_/C_32_ diglycerols detected were bound to a phosphoglycerol (PG) head group at one side of the membrane-spanning molecule (Fig. 6), with no polar head group attached to the other glycerol moiety. The presence of the PG head group bound to one side of the C_30_/C_30_, C_30_/C_32_, and C_32_/C_32_ diglycerol spanning lipids (Fig. 6) strongly suggests that the biosynthesis of the DA membrane-spanning lipids depends on preformed diacylglycerol lipids, the biosynthesis of a PG phospholipid intermediate (Fig. 6), and a membrane-bound enzyme. These observations suggest that the condensation reactions resulting in the formation of membrane-spanning lipids take place when the membrane lipid bilayer is already in place.

We also investigated the membrane lipid biosynthetic pathways in the genome of T. maritima (Table 4). The acylation of the bacterial G3P with specific FA is mediated by the activity of the PlsX/PlsY acyltransferase system followed by the PlsC acyltransferase, giving rise to the phosphatidic acid intermediate (PA) (Fig. 6). Subsequently, PA is activated by CDP-DAG synthase (CdsA) and transformed into glycerol-phosphate by the activity of the PgsA enzyme, converting PA to phosphatidylglycerol phosphate (PGP). Finally, the removal of the inorganic phosphate (P_i_) (probably by an unidentified phosphatase) would lead to the anticipated intermediate for the membrane-spanning IPLs, i.e., the PG phospholipid (Fig. 6). It is conceivable that through these steps the PG phospholipid could be synthesized *de novo*, and the two FA molecules of C_16_ (or C_14_) required for the synthesis of DAs would be incorporated into the glycerol backbone (Fig. 6), leading to C_32_/C_32_ with the PG head group (Fig. 1 and 6) on one side of the compound. Similarly, the FAs attached to the diglyceride backbone found on the opposite side of the membrane-spanning PG compound could be formed through the mentioned steps forming the PA with the diacylglycerol (DAG) precursor, followed by the consequent removal of the phosphate group by a phosphatase. Other metabolic pathways could lead to the formation of the DAG compounds in the cell (26), but it is possible that the IPL glycolipids found on T. maritima also serve as the precursor of DAG prior removal of the Glu or diGlu head group.

**TABLE 4:** Annotated genes in the biosynthetic pathway for glycerophospholipids in T. maritima

Biosynthetic step	Gene name	Gene annotation in T. maritima
Produce G3P from DHAP	*gpsA*	Tmari_0376/Tmari_1438
Diacylglycerol to PA	*glpK*	Tmari_0404/Tmari_0785/Tmari_0954
1st acylation-acyl phosphate	*plsX*	Tmari_0147
1st acylation-acyltransferase	*plsY*	Tmari_1453/Tmari_1289
2nd acylation	*plsC*	Tmari_1701
Activation for head group synthesis	*cdsA*	Tmari_1404
Produces PGP	*pgsA*	Tmari_1876
Dephosphorylates PGP	Phosphatase	Unknown
Coupling of FA which are attached to one PG backbone and one DAG	Membrane-spanning lipid synthase	Unknown
Conversion of ester to ether bonds in the IPL compounds	Modified-*plsA*	Tmari_0479

The anionic IPL PG is known to be one of the principal membrane components of bacteria and is required for its proper function and stability (27). Thus, its formation and distribution across the surface are essential for bacterial survival and cellular organization. The data provided by our study suggest that the hypothetical biosynthetic pathway to produce complex DA-containing lipids involves *de novo* formation of PG and might be selective for the anionic head group of PG.

Since the terminal carbon ends of the FAs are known to be embedded in the hydrophobic core of the membrane, we hypothesize that the enzymatic process implicated in this coupling reaction is catalyzed by a membrane protein that can easily bind or have access to the lipid substrate. Moreover, our finding on the monomethyl diacids and the increased ratio for the C_32_ diacids compared to the C_30_ diacids (Fig. 3B), together with the low abundance of the C_30_ compounds, suggests that the enzymatic activity leading to the membrane-spanning lipids is selective in terms of substrate requirements (i.e., chain length and head group).

### Glycerol ether lipid formation.

As cultures reached the stationary phase, a slight increase in the amount of hydrolysis-derived ether bonds was detected at optimal growth conditions (from 4% to 7%) (Table 1). In contrast, growth experiments at suboptimal temperature showed that the percentage of ether bonds was slightly higher at ca. 10% and remained constant across growth phases. This may represent an adaptation to low temperatures, although typically, the presence of ether bonds in membrane lipids is interpreted as an adaptation to high temperatures, since ether bonds are thought to be more stable than ester bonds (28). In the case of T. maritima, however, these could be the result of adaptation to stressful growth conditions as its optimal temperature of growth is 80°C, while no growth below 55°C is detected (18).

The IPLs detected across growth phases under both temperature conditions showed that the fractional abundance of the sum of IPLs with DAG cores decreased from the early exponential to the stationary phase. In contrast, the IPLs with DEG core lipids showed an apparent increase in their fractional abundance. The mixed cores containing AEG, DAG/DEG, triether, and triester combinations remained constant at 80°C, while at 55°C they increased. Taking these data together, we can speculate that the concomitant decrease on DAG (ester) cores with the increase on DEG (ether) cores is the result of a growth phase-dependent synthesis of ether lipids from ester compounds and that ester lipids can be subsequently converted into ether lipids. Although it is not possible to precisely attribute the changes between ester and ether in the mixed cores to confirm the growth phase dependence for ether lipid formation, our results indicate that the synthesis of ether lipids are not a requirement to make membrane-spanning DAs, as we were able to detect mixed DAG/AEG, DAG/DAG, and DAG/DEG cores in the membrane-spanning PGs across growth phases.

It appears that a considerable part of membrane lipids of T. maritima contains ether bonds in their structure. The strong similarity in the distribution of ether- and ester-derived lipids (e.g., Fig. 3B) suggests that a biochemical mechanism exists that can convert ester bonds into ether bonds. Genes encoding enzymes involved in the biosynthesis of FA-derived ether lipids in bacteria have been described in *Myxobacteria* (29) and potential homologs identified in members of SD4 *Acidobacteria* (11). This set of enzymes is indeed capable of converting an *sn*-1-bound alkyl ester into an ether-bound alkyl moiety. However, homology protein BLAST searches did not result in the detection of close homologs in the genomes of T. maritima and other *Thermotogales* species capable of ether lipid production (7).

Recently, a biosynthetic pathway for the biosynthesis of plasmalogens (i.e., *sn*-1 vinyl ethers) has been identified in the strict anaerobe Clostridium perfringens, consisting of a two-gene operon, *plsA* and *plsR* (30). The proposed mechanism converting the diacyl precursor to a plasmalogen involves two sequential electron reductions, followed by protonation and dehydration (see Fig. S3 in the supplemental material). We searched in T. maritima for potential homologs of *plsA-plsR* from C. perfringens and identified the Tmari 0479 gene as a close homolog but with a different protein domain architecture (Fig. S3). We also identified homologs of a modified *plsA* gene in the genomes of Desulfatibacillum alkenivorans, *Thermodesulfobacterium geofontis*, and “*Candidatus* Kuenenia stuttgartensis” (Fig. S3). These four bacterial species have in common with T. maritima that they produce ether lipids but not plasmalogens, suggesting that the modified PlsA encoded by the Tmari 0479 gene is capable of converting an *sn*-1 ester bond to an *sn-*1 ether bond, potentially with a plasmalogen as an intermediate. In support of this hypothesis, no homologs to this gene could be detected in members of the *Thermotogales* (e.g., *Thermosipho* spp.) that are able to produce tetraester compounds but not mixed ethers/esters lipids (7). Based on this, we hypothesize that the membrane-spanning and FA-derived glycerol ether lipids in *Thermotoga* are synthesized by enzymatic activities that are not related to those of *Myxobacteria* and *Acidobacteria* but rather by a novel mechanism involving a modified *plsA* homolog (Fig. 6). Future work is required to confirm if the enzymatic activity of the modified *plsA* gene product leads to conversion of the ester moiety into a saturated ether in the membrane-spanning lipids of *Thermotoga*. Furthermore, our analysis of the IPLs of T. maritima (Table 2) also reveals that ether bonds do occur at the *sn*-2 position. It remains enigmatic how these are formed.

### Cell profile modification across growth phases and changes at the lipid level.

T. maritima is characterized by an unusual cell envelope, the toga, distending from the poles of the cells by the continued growth of the outer envelope as cells enter the stationary phase under physiological conditions (31). The toga was not visible in the cells grown at optimal temperature (80°C) (Fig. 5). In contrast, cells incubated at 55°C showed a fluorescent membrane signal attributed to the inner and the outer membrane (Fig. 5). Under both examined conditions (optimal and suboptimal temperatures of growth), the cell area and length were significantly reduced by 0.4 and 0.8 μm^2^ and 0.5 and 0.6 μm, respectively, in stationary phase, as previously observed (18). At optimal temperature of growth, the cells become coccoid in shape in stationary phase and increased their circularity values, while at suboptimal temperature the rod-shaped morphology was dominant. Lastly, the membrane permeability was evaluated by assessing the fluorescence measurements of permeable (DAPI) and nonpermeable (SYTOX green) DNA dyes. This analysis revealed that most of the exponential- and stationary-phase cells growing either at optimal or suboptimal temperatures showed fluorescent signals for both DNA dyes. At suboptimal temperature, the fluorescence intensity for both dyes increased significantly in the stationary phase (Fig. 5, Table 3, Fig. S2). This increase at suboptimal conditions suggests these cells have increased membrane permeability relative to those grown under optimal growth conditions. Considering that at both temperatures and stationary phase of growth higher content of membrane-spanning lipids was detected while differences in morphology and membrane permeability were observed, it is possible that other membrane components (e.g., proteins) are involved in the adaptation to different growth temperatures.

### Conclusions.

Cultivation experiments combined with membrane lipid and microscopy analysis identified several environmental and physiological factors affecting the biosynthesis of membrane-spanning lipids in Thermotoga maritima. Our core and intact polar lipid analysis have demonstrated that the synthesis of membrane-spanning lipids across growth phases is dependent on the available fatty acid substrate, and that membrane-spanning lipids increased in stationary phase of growth with independence of temperature. Only intact polar lipids of the membrane-spanning lipid diabolic acid with the polar head group phosphoglycerol were detected. Overall, we have identified the physiological conditions leading to an increase of membrane-spanning lipids in T. maritima, information that is key for further investigations of the nature of their biosynthesis. In addition, we detected a putative coding gene involved in the formation of ether bonds in the membrane-spanning lipids of T. maritima. Taking all the evidence together, we propose a hypothetical biosynthetic pathway for the formation of membrane-spanning lipids of T. maritima that needs to be confirmed by future studies.

## MATERIALS AND METHODS

### Strains, media, and growth conditions.

Thermotoga maritima (MSB8; DSM 3109) (18) was obtained from the DSMZ culture collection and was cultivated under anaerobic conditions in 120-ml batch cultures in 250-ml serum bottles. The basal medium (BM) (32) used was composed of (per liter) 1 g NH_4_Cl, 0.3 g KH_2_PO_4_, 0.3 g K_2_HPO_4_, 0.1 g KCl, 25 g NaCl, 6.5 g MgCl_2_·6H_2_O, 0.13 g CaCl_2_·2H_2_O, 1 g yeast extract, 10 ml Balch’s trace mineral solution (33), 0.5 mg resazurin, and pH adjusted to 7 with KOH. The medium was anaerobically dispensed in 250-ml serum bottles, and a gas phase of N_2_-CO_2_ (80:20, vol/vol) was applied. After sterilization, individual bottles were supplemented with 21 mM NaHCO_3_, 2.1 mM Na_2_S·9H_2_O, 18.7 mM glucose, 2.5 mM l-cysteine hydrochloride monohydrate. Cultures were first grown at 55 or 80°C for three subcultivation growth cycles to ensure acclimatization. The growth phase experiment was inoculated during the fourth subcultivation at the mentioned temperature. Growth was monitored by measuring the optical density at 600 nm (OD_600_), and cells were harvested at the early exponential (OD_600_, 0.1), exponential (OD_600_, 0.2), and stationary (OD_600_, 0.3 to 0.4) growth phases. The experiments were performed in triplicate.

### Lipid extraction and analysis.

For lipid analysis, cell material from 120 ml culture was collected by centrifugation and freeze-dried, and biomass was harvested at each growth phase and temperature condition, split in equal amounts, and subjected independently to core and intact polar lipid (IPL) analysis.

For core lipid analysis, the freeze-dried cell pellet was base hydrolyzed with 2 ml of 1 N KOH in MeOH solution and refluxed for 1 h at 130°C, after which the pH was adjusted to 4 to 6 with 2N HCl solution. Phase separation was achieved by the addition of 2 ml dichloromethane (DCM) and 2 ml bidistilled water, and the organic layer was removed. The aqueous layer was washed one more time with DCM. DCM layers were combined and dried over Na_2_SO_4_ columns. Before analysis, the extracts were treated with pyridine (10 μl) and N,O-bis(trimethylsilyl)trifluoroacetamide (BSTFA) (10 μl) to derivatize alcohol groups and then brought to a final volume with ethyl acetate to produce a concentration of 1 mg ml^−1^. Core lipid quantification (relative abundance) was carried out on an Agilent Technologies 7890B GC with a silica column (CP Sil-5; 25 by 0.32 mm) and analyzed under conditions described previously (7). Core lipid identification was carried out on an Agilent Technologies 7890A GC coupled to an Agilent Technologies 5975C VL MSD mass spectrometer (MS) operated at 70 eV, with a mass range *m/z* 50 to 600 and 3 scans s^−1^. The column and oven settings were the same as those for the quantification GC analysis. Core lipids were identified based on literature data and library mass spectra.

IPL analysis was performed on single samples obtained from each temperature and growth phase condition. IPLs were extracted from the freeze-dried biomass from different growth phases using a modified Bligh and Dyer procedure (34) and were analyzed by high-performance liquid chromatography-ion trap mass spectrometry (HPLC-ITMS) as described previously (35). IPLs were quantified in terms of their MS peak area response. The peak areas were determined from extracted ion chromatograms of either the [M + H]^+^ or [M + NH_4_]^+^ ion, or combinations as appropriate, for each IPL species. The relative abundance of peak areas does not necessarily reflect the actual relative abundance of the different compounds; however, this method allows for comparison between the samples analyzed in this study. To confirm IPL identification using accurate masses, additional analyses on selected samples were carried out using an ultrahigh-performance liquid chromatography-high-resolution mass spectrometry (UHPLC-HRMS) according to the reverse-phase method of Wörmer et al. (36) with the same UHPLC-HRMS system operated with the same settings as those described in Bale et al. (35).

### Statistical analysis.

To assess whether the core lipid changes were statistically significant, a two-way ANOVA with Tukey’s multiple-comparison test was conducted on the mean relative abundance of each detected core lipid analyzed in triplicate. A *P* value of <0.05 was considered statistically significant. Data are presented as means ± standard errors of the means. To assess whether the cell measurements were statistically significant, one-tailed Student's *t* test was conducted on a mean of 100 analyzed on 3 independent images. A *P* value of <0.05 was considered statistically significant. Data are presented as means ± standard deviations. The analysis was conducted using GraphPad Prism Software version 9.02 (GraphPad Software, San Diego, CA).

### Genomic analysis.

The genes coding for the glycerophospholipid biosynthetic pathway were retrieved from KEGG-based annotations (37), and the identifiers were obtained from UniProt (38). Searches for biosynthetic genes in T. maritima MSB8 homologous to the *elbD* gene of the ether lipid pathway were performed with the PSI-BLAST algorithm (position-specific iterated BLAST) (39) at the protein level using (Q1DC43) from Myxococcus xanthus DK 1622 as the query. Similarly, the *plsA* genes for the ether lipid pathway of T. maritima MSB8 DSM 3109, Desulfatibacillum alkenivorans PF2803, “*Ca.* Kuenenia stuttgartensis,” and *Thermodesulfobacterium geofontis* OPF15 were identified with the PSI-BLAST algorithm using the *plsA*-*plsR* operon (Q8XL48 and Q8XL47) from Clostridium perfringens strain13 and *plsA* (Q835P9) from Enterococcus faecalis V583 as the query.

### Assessment of cell morphology and membrane viability by fluorescence microscopy and image analysis.

To visualize cells, 1 ml of actively growing culture was anoxically sampled and collected by centrifugation (10 min by 13,000 rpm) and washed with 1 ml of anoxic BM medium. Cells were then resuspended in 1× staining buffer containing the membrane dye FM4-64 (1 μg/ml), the membrane-permeable DNA stain 4′,6-diamidino-2-phenylindole dihydrochloride (DAPI) (2 μg/ml), and the membrane-impermeable DNA stain SYTOX green (0.5 μM), and 5 μl of concentrated cells was transferred to an agarose pad (0.5% agarose). Cells were visualized on an Axioplan 2 imaging microscope (Carl Zeiss, Germany) coupled to an AxioCam MRc5 camera, a 100×/1.30 oil immersion objective, and a Colibri LED light source. The microscope was controlled with AxioVision 4.8 software (Carl Zeiss), and the fluorescent images were captured with appropriate filter sets from Zeiss (02 for DAPI, 38 for SYTOX green, and 09 and 00 for FM4-64). The microscopy images were acquired with DAPI and SYTOX green dye independently within the same visualization area to obtain the fluorescence data. To get the quantitative data, the images were analyzed with ImageJ using the MicrobeJ plug-in, which detects objects in the image by utilizing the morphology with associated parameters. The analysis was performed on the entire image, from which 100 cells that met the morphology attributes were randomly selected for retrieving the data utilized in the statistical analysis and comparisons. In our approach, the cell measures (area, length, and circularity) are represented by the data obtained with DAPI, which records the nucleoid area. In the case of the fluorescence intensity values, they were obtained from both independent fluorescent images (DAPI and SYTOX green).

### Data availability.

The data sets supporting the conclusions of the study are included in the main text and figures/table and in the supplemental material.
