# Supplementary material for: Changes in the Distribution of Membrane Lipids during Growth of Thermotoga maritima at Different Temperatures: Indications for the Potential Mechanism of Biosynthesis of Ether-Bound Diabolic Acid (Membrane-Spanning) Lipids
Source: Appl Environ Microbiol. 2022 Jan 25;88(2):e01763-21. doi: 10.1128/AEM.01763-21 (PMC8788747; doi:10.1128/AEM.01763-21)
Supplement: Supplemental file 1 — Table S1, Fig. S1 to S3. Download AEM.01763-21-s0001.pdf, PDF file, 0.4 MB [file aem.01763-21-s0001.pdf]

Supplementary Table 1. IPLs with decanoyl-diglucosyl headgroups; accurate masses and their fragments (UHPLC-HRMS MS^2).

Core	*m/z* of $[M+NH_4]^+$	AEC	Δ mmu*	*m/z* of fragment	AEC	Δ mmu*	Rationale
DAG, $C_{32:0}$	1064.783	$C_{57}H_{110}NO_{16}$	-0.6	551.504	$C_{35}H_{67}O_4$	0.1	DAG $C_{32:0}$ core (loss of head group)
				313.274	$C_{19}H_{37}O_3$	-0.1	$C_{16:0}$ FA+ glycerol
				317.196	$C_{16}H_{29}O_6$	-0.2	Hexose + C_{10} FA
DAG, $C_{30:0}$	1036.751	$C_{55}H_{106}NO_{16}$	-0.6	523.472	$C_{33}H_{63}O_4$	-0.4	DAG $C_{30:0}$ core (loss of head group)
				313.273	$C_{19}H_{37}O_3$	-0.4	$C_{16:0}$ FA + glycerol
				285.242	$C_{17}H_{33}O_3$	-0.2	$C_{14:0}$ FA + glycerol
				317.196	$C_{16}H_{29}O_{61}$	-0.2	Hexose + C_{10} FA
AEG, $C_{32:0}$	1050.803	$C_{57}H_{112}NO_{15}$	-0.5	537.524	$C_{35}H_{69}O_3$	0.2	AEG $C_{32:0}$ core (loss of head group)
				313.273	$C_{19}H_{37}O_3$	-0.3	$C_{16:0}$ FA + glycerol
				317.196	$C_{16}H_{29}O_6$	0.3	Hexose + C_{10} FA
AEG, $C_{30:0}$	1022.772	$C_{55}H_{108}NO_{15}$	-1.0	509.493	$C_{33}H_{65}O_3$	0.1	AEG $C_{30:0}$ core (loss of head group)
				313.274	$C_{19}H_{37}O_3$	-0.2	$C_{16:0}$ FA + glycerol
				317.196	$C_{16}H_{29}O_6$	0.0	Hexose + C_{10} FA

DAG = diacylglycerol; AEG = mixed acyl/etherglycerol; * (Measured mass – calculated mass) x 1000; AEC = assigned elemental composition

Supplementary Figure 1. Fluorescence images of *T. maritima* cells from stationary cultures after prolonged incubation at optimal growth temperature (80°C) Cell shape assessment of *T. maritima* cells after one-week incubation at optimal temperature. Membrane stain FM4-64 (red) DNA stains: Membrane permeable; DAPI (blue) membrane-impermeable; SYTOX (green) Scale bar, 5 μm

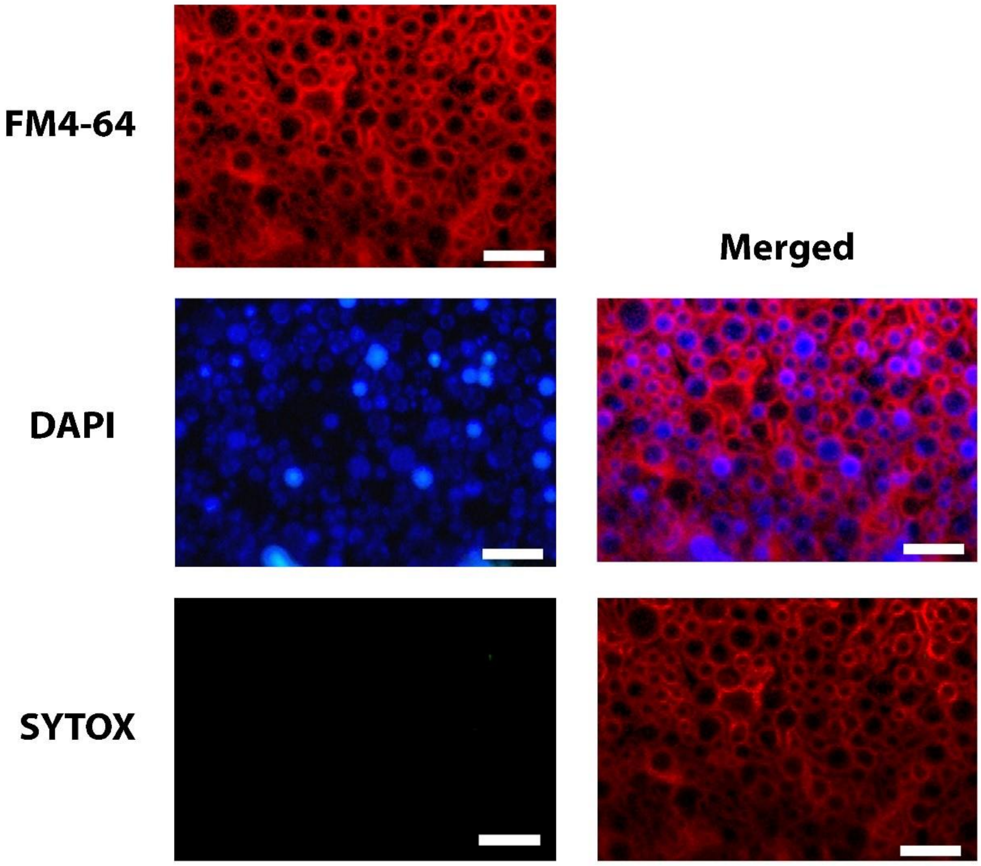

Supplementary Figure 2. Cell measurements from fluorescent microscopy analysis Data represent the mean with SD ±, n = 3 independent images with 100 cells assessed. Student's t-test was carried out on the mean of 100 cells per triplicate from each condition to determine the statistical significance of the differences of means between A) growth phases and B) temperature conditions A p-value < 0.05 was considered statistically significant (one-tailed Student's t-test), n.s. indicates no significant

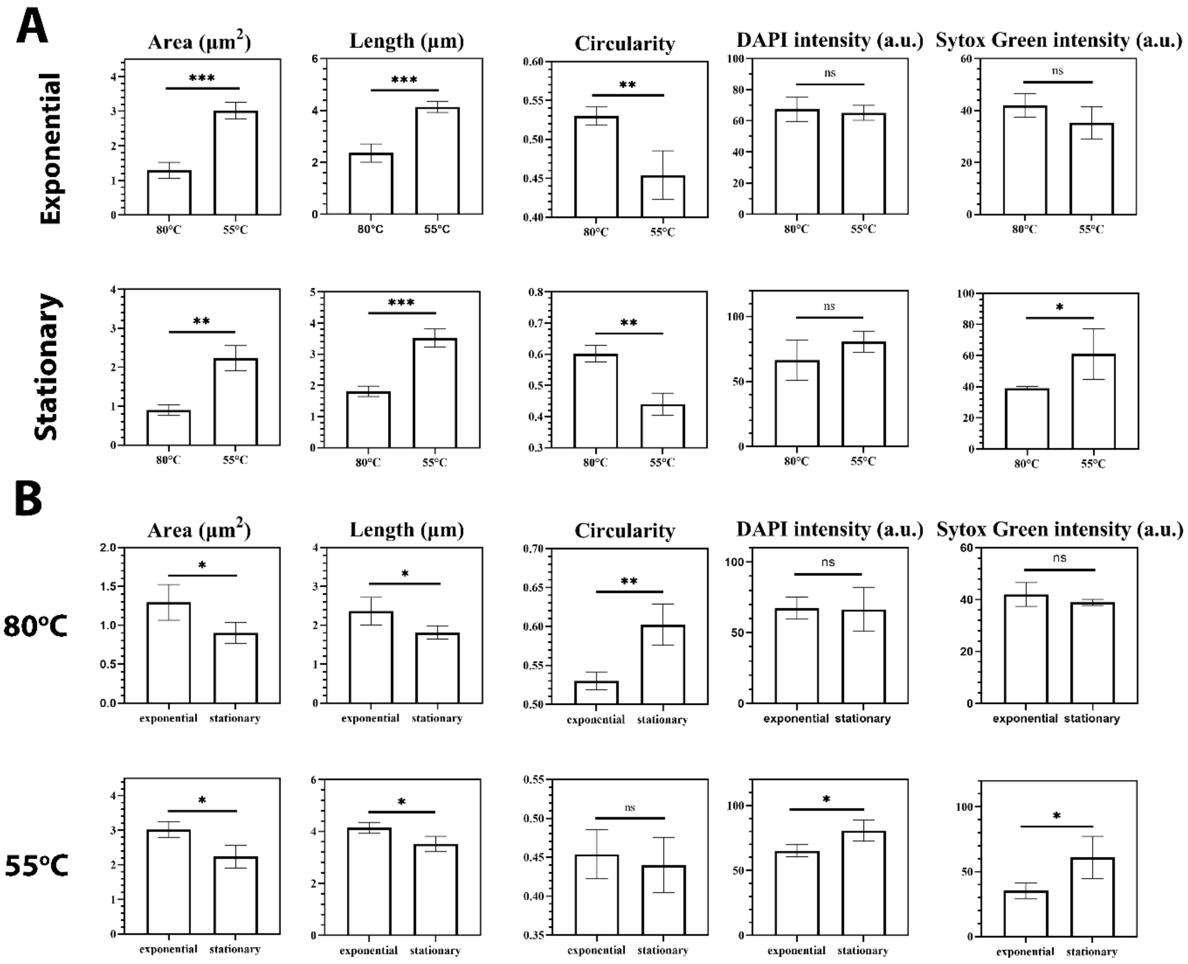

Supplementary Figure 3. Predicted domain analysis of plasmalogen and ether lipid proteins. The distribution of homologous genes to the *Clostridium perfringens* plasmalogen biosynthetic pathway across obligate and facultative anaerobic bacteria as *Enterococcus faecalis* revealed three distinct architectures containing the Pfam domains. The plsA/plsR operon (CPE1194 and CPE1195) from *C. perfringens* and the plsA single protein (EF_1327) from *E. faecalis* are composed of two activation domains (Benzoyl-CoA reductase PF01869+PF01869) and two-electron transfer domains (a CoA enzyme activase uncharacterized domain PF09989+ 2-hydroxyglutaryl-CoA dehydratase, D component F06050). In bacteria known to be able to synthesize ether lipids but not plasmalogens (i.e., unsaturated ethers) such as *Thermotoga maritima*, *Desulfatibacillum alkenivorans*, *Thermodesulfobacterium geofontis* and *Ca. Kuenenia Stuttgartensis*, the activation domains (PF01869+PF01869) and the electron transfer domain are conserved (PF09989). The second reduction/dehydration domain is replaced by four small functional domains (PF06050+PF09989+PF09989+PF06050). PF01869+PF01869+PF09989 architecture is conserved in the protein encoded by the *T. maritima* Tmari 0479 gene, but instead of the ultimate PF06050 domain, it contains four smaller functional domains, PF06050+PF09989+PF09989+PF06050 along the protein sequence. In the case of *T. geofontis*, the PF09989+PF09989 domains, which each comprise ca. 50 amino acids per domain in the other mentioned species, is replaced by a larger PF09989 domain comprising ca. 220 amino acids. Analysis performed with Interpro scan (1) and Pfam (2). Amino acid position of the predicted functional domains are indicated above each box (showing the PF domain). Figure created using BioRender (https://biorender.com/).

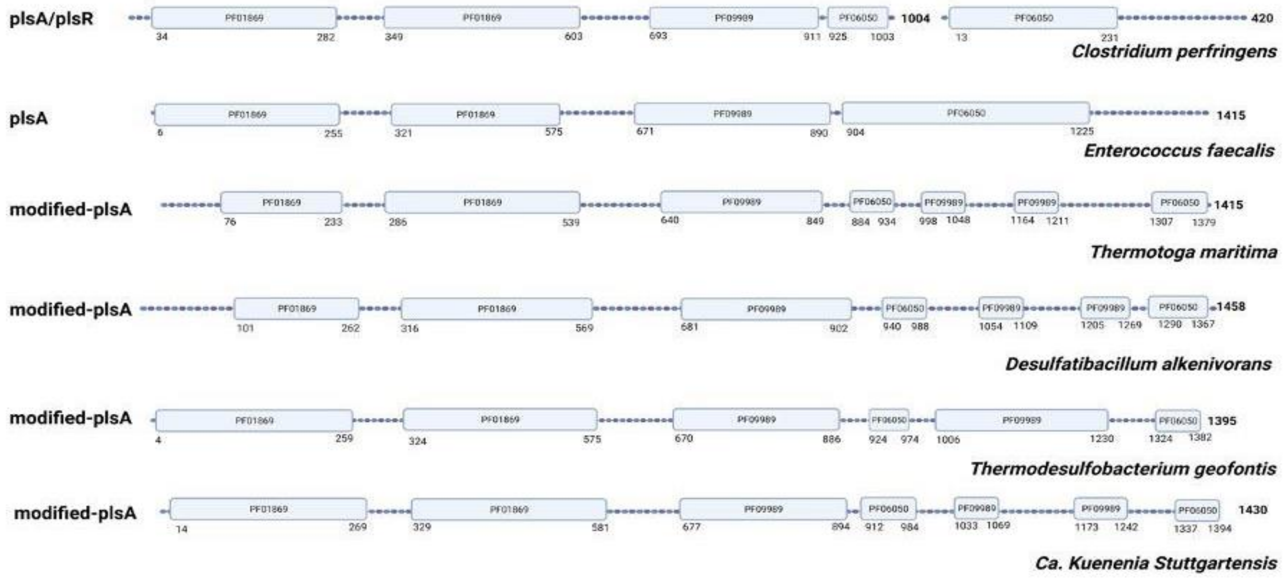

References

1. Hunter S, Apweiler R, Attwood TK, Bairoch A, Bateman A, Binns D, et al. 2009. InterPro: The integrative protein signature database. Nucleic Acids Res 37:D211–D215.

2. Finn RD, Bateman A, Clements J, Coggill P, Eberhardt RY, Eddy SR, et al. 2014. Pfam: The protein families database. Nucleic Acids Res 42:D222–D230.
